# Digitalization enhancement in the pharmaceutical supply network using a supply chain risk management approach

**DOI:** 10.1038/s41598-023-49606-z

**Published:** 2023-12-15

**Authors:** Wai Peng Wong, Pui San Saw, Suriyan Jomthanachai, Leong Seng Wang, Huey Fang Ong, Chee Peng Lim

**Affiliations:** 1 School of Information Technology, Monash University Malaysia, 47500 Selangor, Malaysia; 2 School of Pharmacy, Monash University Malaysia, 47500 Selangor, Malaysia; 3 Faculty of Management Sciences, Prince of Songkla University, Songkhla, 90110 Thailand; 4 Institute for Intelligent Systems Research and Innovation, Deakin University, Geelong, Australia

**Keywords:** Risk factors, Quality of life, Computational science

## Abstract

One major issue in pharmaceutical supply chain management is the supply shortage, and determining the root causes of medicine shortages necessitates an in-depth investigation. The concept of risk management is proposed in this study to identify significant risk factors in the pharmaceutical supply chain. Fuzzy failure mode and effect analysis and data envelopment analysis were used to evaluate the risks of the pharmaceutical supply chain. Based on a case study on the Malaysian pharmaceutical supply chain, it reveals that the pharmacy node is the riskiest link. The unavailability of medicine due to unexpected demand, as well as the scarcity of specialty or substitute drugs, pose the most significant risk factors. These risks could be mitigated by digital technology. We propose an appropriate digital technology platform consisting of big data analytics and blockchain technologies to undertake these challenges of supply shortage. By addressing risk factors through the implementation of a digitalized supply chain, organizations can fortify their supply networks, fostering resilience and efficiency, and thereby playing a pivotal role in advancing the Pharma 4.0 era.

## Introduction

Supply shortages in the pharmaceutical industry, as highlighted in a recent study conducted by^1^, are a significant concern with far-reaching consequences. Not only does it affect individual health outcomes, but it also affects the broader healthcare system as well^2^. While patients who rely on consistent access to essential medications face uncertainty and potential health risks, healthcare providers and institutions are burdened with the challenge of managing and mitigating the impact of these shortages on patient care^3^. In addition, the economic implications are substantial, as they contribute to increased healthcare costs as healthcare providers seek alternatives, which are often more expensive, or incur additional costs related to managing patient health complications arising from medication unavailability^4,5^. These disruptions in the availability of critical medications underscore the need for a closer examination of the root causes^6^. Factors such as manufacturing issues, regulatory challenges, and complex global supply chain dynamics contribute to these shortages, making it essential to investigate comprehensively and implement effective solutions^4,5^.

In fact, pharmaceutical supply issues have long been a persistent and significant issue within the global healthcare system^7^. This stems from the complexities of the pharmaceutical supply chain which its characterisitcs are different from other industries. One example of the characteristics is it is fragmented and involve many different stakeholders. Addressing drug supply challenge requires a concerted effort from all stakeholders involved, as emphasized by^8^, without seamless supply coordination, a sudden demand surge could strain supply chain networks and worsen disruptions. Some scenarios that are causing *supply issues* are such as the misallocation of medication resulting from an increased demand for therapeutic supply could exacerbate drug shortages in community pharmacies^9^. Ref.^10^ also reported that healthcare policymakers are constantly grappling with delays and non-fulfillment of medication orders, leading to widespread drug shortages. While the current systems face operational, logistics, and infrastructure challenges, several measures can be implemented to significantly ease the strain and address the issue associated with drug or supply shortages^11^.

Pharma 4.0, also known as the fourth industrial revolution in pharmaceutical manufacturing, is characterized by the integration of advanced technologies such as artificial intelligence (AI) and the Internet of Things (IoT) into the manufacturing process. Pharma 4.0 offers robust and flexible manufacturing processes, less interruption in medicine production and delivery, increased productivity, improved connectivity, and a fast response to drug or supply shortages. As a result, Pharma 4.0 can ensure better clinical and operational performance^11^. Pharmaceutical supply chain processes are critical to improving the overall performance in the Pharma 4.0 era. In this respect, organizations should consider redesigning their traditional business models by adopting a digital supply chain to achieve operational effectiveness and manage disruption^12^.

In this paper, we aim to address two primary research questions: 1) What are the most significant risks factors in the pharmaceutical supply chain in Malaysia that affect supply shortages? How can these risks be mitigated? Therefore, the objective of this study is firstly to evaluate and identify the siginifcant risks factors in the pharmaceutical supply chain. Secondly, to propose an appropriate digital technology platform to address these risks. The contributions of this study are twofold. First, it proposed a risk management approach for risk assessment in the pharmaceutical supply chain. Second, the derivation of managerial insights with a proposed research framework to incorporate and encourage digitalization of the pharmaceutical supply chain. The organisation of this paper consists of 6 sections, namely (i) an introduction; (ii) a literature review on the pharmaceutical supply chain and risk factors and emerging digital-based technologies in the pharmaceutical supply chain; (iii) methodology (iv) results, analysis and discussion; (v) managerial implications; (vi) and conclusion.

## Literature review

### Pharmaceutical supply chain and factors contributing to the risks

A pharmaceutical supply chain (PSC) with optimum operational performance is essential for efficient delivery of medications to the patients. This can be measured by 5 different aspects, which are cost, quality, delivery, flexibility and dependability^13^. However, PSC is much more complex compared to other industries, considering the products are potentially life-saving for the patients and it has to be accurately and adequately provided to suit the needs of patients, not to mention that the industry has been advancing to personalised and patient-specific medications^14–17^. PSC covers a widespread network, often extending to other countries, that involves a plethora of stakeholders.

**Figure 1:**
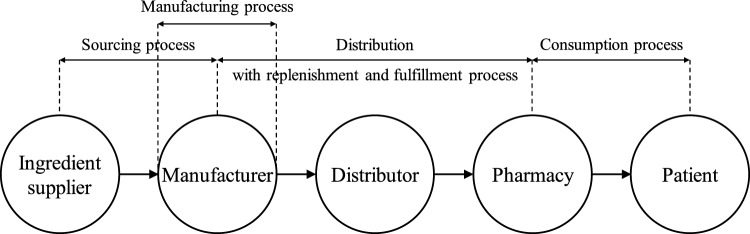
Pharmaceutical supply chain and the main processes, modified from Saha et al.^11^ and Musamih et al. (2021).

Referring to Fig. 1, PSC can be segmented into three distinct levels, which are upstream (sourcing), central (distribution) and downstream (consumption) respectively. The sourcing process can loosely be defined as the manufacturers and the importers; the distribution process includes the wholesalers or distributor; while the consumption process is composed of the pharmacies (i.e., hospital pharmacies, clinics, community pharmacies) as well as the end users which are the patients.

The manufacturing and distribution processes have to be reliable, responsive and flexible in adapting to the demands of pharmaceutics, which are often time unpredictable by nature^18^. This is because it is not only affected by external factors such as political, social and economic status, but also highly dependent on consumer factors and drug factors^19^. As for consumer factors, other stakeholders such as prescribers, pharmacists and payers are most of the time the main decision-makers of consumption rather than the patients themselves, hence the difference in practices and policies could complicate the forecasting of demand. The competition within the pharmaceutical market also varies from drug to drug as the replaceability of a therapeutical product depends on patents, availability of generic products and clinical evidence^20^. Given that PSC is inherently associated with these complex properties, inefficiency of operational performance is bound to occur within PSC, which poses varying degree of disruption risks to the PSC.

### Factors contributing to the risks

The factors that contribute to this are interrelated to properties of PSC described above. Firstly, the large number of stakeholders involved in the fragmented PSC has led to disconnections and in turn lack of accountability between the supply chain partners, where information is not transparent across the intermediaries and the multiple consumption points^17,21,22^. Secondly, the lead time in PSC is usually long due to the time needed for the processes at each level of the PSC, especially when there is a need to fulfil the regulatory requirement^18^. As such, any changes in the demand at downstream, which is unpredictable in the first place, could cause a phenomenon known as “bullwhip effect”, where there would be a large fluctuation in quantities required to be supplied at the upstream level, hence the demands may not be met^21,22^. Financially, inaccuracy in demand forecast could lead to losses due to declining of sales if the amount of stock is inadequate. Therefore, high operating costs is required to maintain an optimum inventory level and warehouse spaces. However, this comes with a few downsides as it also affects the cash flow by prolonging the cash-to-cash cycle time and may potentially cause wastage due to damages and expiration of the products.

Besides the risks associated with operation and inventory management, different modes of transportation are also associated with their respective risk which could occur during the shipment preparation, storage and transportation process and lead to delay in delivery, damage to the goods and temperature excursion^23^. Crises of various foreseeability like natural disasters, political instability and pandemics can impact every stage of the supply chain, leading to different magnitudes of disruptions, damages and losses^24,25^. Lastly, regulatory issues such as documentations including licensing and permits, bureaucracy, changes in regulatory standards and drug recalls are major risks with high severity^26,27^.

In a nutshell, the pharmaceutical supply chain's distinct vulnerability to disruptions and risks, encompassing factors such as demand uncertainty, operational inefficiencies, inventory management, transportation challenges, and regulatory compliance, can significantly impede production and disrupt the seamless flow of drug/medication products, ultimately resulting in supply shortages within the pharmaceutical supply chain^28^. Shortages of essential medicines not only harm patients but also have a significant impact on the economy^29^. Drug shortages present a multi-dimensional challenge^30^. In-depth investigation in the local context is therefore crucial for determining the root causes of supply/medicine shortages and the complex interplay between various factors such as supply chain logistics, regulatory policies and manufacturing processes^11^.

In Pharma 4.0, manufacturing processes can become more robust and flexible, which could result in fewer interruptions in medicine production and delivery, better connectivity, faster responses to drug shortages, and increased productivity. Ref.^11^ also highlighted that Pharma 4.0 could improve clinical and operational performance. To highlight the challenges in the pharmaceutical supply chain, the following sub-sections examine emerging digital-based technologies in the Pharma 4.0 supply chain.

### Emerging digital-based technologies in the pharmaceutical supply chain

Studies on the emerging digital-based technologies in the pharmaceutical supply chain in Google Scholar (period of 2011–2022) are reviewed and summarized in Table 1. Specific contributions of each technology were stated in the third column of Table 1.

**Table 1:** A summary on the emerging digital-based technologies in the pharmaceutical supply chain.

Process	Digital-based technologies	Contribution of each technology/Function application	Reference
Sourcing	Big data analytics	Analysis of data for supplier evaluation and selection based on historical performance and risk data to manage suppliers more effectively	^31,32^
	Cyber-physical system (CPS)	Guarantee the just-in-time delivery to safety stocks, and facilitate the production of suppliers	^32^
	Blockchain	Verifying each and every vendor and their respective raw material quality from the source of origin	^33^
Manufacturing	Automation/sensors	Increase efficiency and quality which can mitigate human instability errors and control environmental factors more effectively	^32^
	Process Analytical Technologies (PAT)	Used to plan, analyze, and control production processes to fulfill the real-time release demand and online quality monitoring	^32,34^
	Blockchain	Merged data for each and every facility will be more accurate and trusted with continuously monitor and track the drug transfer process	^33^
		Manage certifications and government regularities	^33^
	Big data analytics/machine learning	Reflect potential machine failures and conduct proactive maintenance	^32^
	3D printing	Offer solutions for flexible small scale production or customized manufacturing	^32,35^
Distribution	CPS/Internet of Things (IoT)/cloud computing	Provide a high level of connection and traceability for both information flow and physical flow	^32^
	Automatic identification (Auto-ID)	Supply chain visibility (SCV) of good distribution practice	^36^
	Big data analytics	Assist strategic level decision-making, the storage location, route of delivery and operational performance	^32,37^
	Blockchain and IoT integration	Traceability logistics for tampering of products, delay, and fraud	^33,38–40^
	Radio frequency identification (RFID) and cloud computing	Track the products in real-time and reduce misplacement	^32,37^
	Autonomous and smart vehicles	Aid distributors to save more operation costs in the delivery and sorting processes, and optimize lead times and ecological impacts	^32,41^
	Drone	Provide flexible and innovative delivery processes	^32,41^
Fulfillment	Big data analytics	Analyze a distribution rate to improve forecasting order of fulfilment	^11^
Replenishment	Cloud and fog computing	Support cloud-based ordering systems, electronic data interchange, online ordering, automated procure-to-pay	^11^
	CPS	Flexible and autonomous replenishment and inventory management	^32^
	E-procurement and open contracting	Promoting anti-corruption, transparency and accountability	^42^
Consumption	Big data analytics	Extract value from challenging amounts of data such as consumption data to plan and forecasting demand	^11,43^
	Machine learning	Recommend the top-rated or best medicines to the customers	^38^
	Blockchain	Detecting falsified and substandard drugs in consumers dispensing	^44–46^

## Methodology

### Methodological approach

Risk management is a systematic process employed by organizations to identify, evaluate, and mitigate potential risks that may impact their operations. It involves the comprehensive analysis of uncertainties and threats, enabling businesses to make informed decisions and enhance their overall resilience. One powerful tool for risk assessment within this framework is Failure Mode and Effects Analysis (FMEA), which systematically examines potential failure modes, their causes, and their consequences^47^. This enables organizations to prioritize and address critical risks, ensuring a proactive approach to risk management and ultimately strengthening their operations.

**Figure 2:**
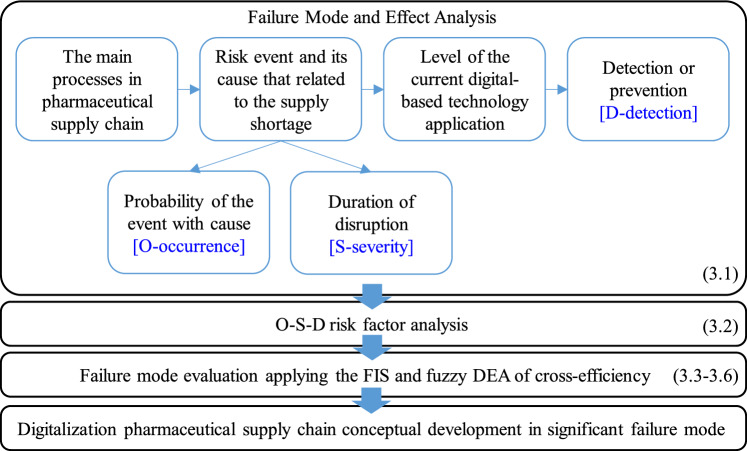
The conceptual methodology of a digitalized supply chain using a supply risk management approach.

Figure 2 depicts the conceptual methodology of this study. The concept begins with the Failure Mode and Effect Analysis approach (FMEA). FMEA was chosen because it is a well known technique used in risk assessment^48,49^. The failure modes associated with the pharmaceutical supply chain are investigated after identifying the main supply chain processes. The main supply chain processes were discussed in detail in section "Risk assessment metric" and summarized in Appendix (Table A). Following the identification of failure modes or risk events, the risks are assessed by calculating the values of three factors: the O (Occurrence) factor, the S (Severity) factor, and the D (Detection) factor. In a real-world setting, we conducted interviews with respondents to ascertain these values. The O-factors are generated by asking, “How often does this event occur?”. The S-factor can be investigated, with the question: “If this failure occurs, how long will it affect the operations?”. The S-factors are developed based on the idea that the severity of a supply shortage can have a varying level of impact on patients concerning different categories of medicine products, increasing the difficulty of analysis. The duration of disruption^50^ is used to investigate this factor in this study. In addition, the D-factors are calculated using the question “How effective is current digital-based technology in detecting or preventing this failure?” For risk analysis, these three O-S-D factors are scaled based on^48^ perspective, as shown in Table 2.

**Table 2:** Risk factor analysis scale.

O-factor		S-factor		D-factor	
“How often does this event occur?”		“If this failure occurs, how long will it affect the operations?”		“How effective is current digital-based technology in detecting or preventing this failure?”	
1	Never	1	Less than a day	1	Very high
2	Less than once per year	2	Average a day		
3	Once per year	3	Less than a week	3	High
4	More than once per year	4	Average a week		
5	More than once per half year	5	Less than a month	5	Moderate
6	More than once per quarter	6	Average a month	7	Low
7	More than once per month	7	Average a quarter		
8	More than once per week	8	Average a half year	9	Very low
9	Once per day	9	Average a year		
10	More than once per day	10	More than a year	10	None apply

Subsequently, the O-S-D risk is assessed. Note: The O-S-D risk factor analysis is presented in section "Risk assessment metric".

To assess the failure mode, given the inherent subjectivity and potential fuzziness in human inputs, we utilized the Fuzzy Inference System (FIS) and Data Envelopment Analysis (DEA). These methods were employed to handle the linguistic variables associated with both inputs and outputs, facilitating the evaluation of results.

The risk assessment metric and failure mode evaluation using the FIS and DEA are explained in sections "Risk factor fuzzification"–"DEA and the cross efficiency method". In this study, as mentioned in prior Sect. (3.1), the risk measure is based on FMEA. Since the traditional FMEA suffers from the uncertainty and ambiguity of expert assessment in real-world environments^51^, we apply the fuzzy approach of FIS to FMEA to overcome this drawback. Then, DEA is employed to calculate the risk based efficiency. DEA is also useful for overcoming the simplified mathematical formula in calculating the risk priority number (RPN) in FMEA, which can provide counterintuitive statistical properties. In contrast, the use of DEA with FMEA can tackle issues such as non-consideration of the direct/indirect relationships between failure modes. Instead of directly applying fuzzy DEA, which uses the fuzzy sets of O-S-D factors as the input of the DEA method, we exploit the FIS to provide fuzzy inputs with a fuzzy rule set based on the significance of O-S-D factors. For this purpose, the rule set comprising different weights of O-S-D factors can be utilised to incorporate expert experience and viewpoint on the identified weights of O-S-D input variables, in order to make the corresponding fuzzy inference^52^. The advantage of employing FIS on the inputs, as opposed to directly applying fuzzy DEA, lies in FIS’s superior ability in handling qualitative or linguistic data. FIS allows for the incorporation of domain knowledge and expert opinions into the model, enhancing the interpretability of the results.

### Risk assessment metric

In this study, the risk assessment metric is developed based on the risk management concept. Firstly, the risk identification process is carried out. Table 3 depicts the findings on risk identification pertaining to supply shortage in the pharmaceutical supply chain, which is based on the respective critical processes. A scheme to analyze the level of risk factors is developed, as shown in the Appendix. Based on the pharmaceutical supply chain in Malaysia, the manufacturer node evaluates the metric related to the sourcing, manufacturing, and order fulfillment processes. The distributor node validates the metric for the distribution, order fulfillment and inventory replenishment processes. The pharmacy shop node also assesses the metric for replenishment and consumption.

**Table 3:** Identification of supply shortage risks in the pharmaceutical supply chain.

Event	Cause of event (failure no.)	Detection or prevention	Mapping to digital technology	Reference
Process: sourcing				
Unavailability of raw materials	Limit or single supplier yield as a material source from a specific source (S1)	Multiple suppliers and multiple countries strategy and supplier risk management	Big data analytics	^5,7,53^
	Political turmoil (S2)	↑	↑	^5,53^
	Armed conflicts (S3)	↑	↑	^5,53^
	Trade disputes (S4)	↑	↑	^5,53^
	Raw materials can become contaminated or degraded during storage or transport (S5)	Tracking and traceability rules	CPS	^54^
Delay in raw material supply	Almost oversea suppliers (S6)	De-globalization strategy	Big data analytics	^54^
Process: manufacturing				
Unable to produce medicines to meet the order	Capacity constraints (M1)	Build a production network to enhance the flexibility and capacity to respond to the emerging demand of medicines	PAT/blockchain	^5,54^
	Limited production capabilities (drug-manufacturing difficulties) (M2)	Build a production network to promote the knowledge transfer process	Blockchain	^53^
	Facilities operation/maintenance in inefficiency (M3)	Improve the effectiveness of the manufacturing processes	Automation/sensors/big data analytics/machine learning	^5,7,54,55^
	Quality problems/voluntary recall of product (M4)	Establish more robust and agile manufacturing processes that have fewer interruptions, less defects, and higher levels of quality management	Automation/sensors/PAT	^5,54,55^
	Just-in-time or lean inventory system of raw material inefficiency/no buffer stock control (M5)	Improve the inventory and control management system	CPS	^5,54^
	Cost raise of raw materials and production (M6)	Increase efficiency of production and quality	Automation/sensors/PAT	^5^
	Low market size/low-profit margin/batch constraints (M7)	Development the solution of flexible small-scale production or customized manufacturing	3D printing	^5,54^
	Poor coordination among the internal departments (M8)	Improve the information transfer process	Blockchain	^5^
Regulatory barriers	Poor communication/cooperation between the Food and Drug Administration (FDA) and manufacturer (M9)	Transparent communication and cooperation system improvement	Blockchain	^54^
Process: distribution				
Unable or delay supply due to transportation issue	Delay in shipment of the drug (D1)	Tracking and traceability rules	CPS/Internet of Things (IoT)/cloud computing/RFID/Auto-ID	^5,7^
	Transportation and distribution facilities breakdown (D2)	Improve the facilities maintenance planning system	Big data analytics/machine learning	^56^
	Transportation disruptions (D3)	Change mode of transport/route	Big data analytics/drone	^56^
Unable or delay supply due to distribution center/warehousing issue	Inefficient storage system (D4)	Conduct regular checks to ensure suitable conditions of light, humidity, ventilation, temperature, and security	Big data analytics/blockchain and IoT integration	^57,58^
	Inefficient/delay operation (D5)	Warehouse operation must be reduced using appropriate methods which automatically set targets	Big data analytics/Automation/sensors/PAT	^32,57^
Process: fulfillment and replenishment				
Unable or delay supply due to poor order fulfillment and inventory replenishment	Just-in-time or lean inventory system of drug inefficiency/no buffer stock control (F1)	Tracking and traceability rules	CPS	^5,54^
	Poorly performed ordering system (R1)	Develop a system purpose for ordering practices	Cloud and fog Computing/CPS/E-procurement and open contracting	^59^
	Unethical/uncontrolled marketing strategies of the manufacturer (i.e. keep stock for increasing price product) (R2)	Intensive control in inventory and allowing stakeholders to know how much product is available	Big data analytics/Blockchain	^5,54^
	Drugs are only produced by a few manufacturers (R3)	↑	↑	^5,54^
Process: consumption				
Unavailability of product	Unpredictable demand (C1)	Independent pharmacies should coordinate and work together, closely to be informed, and sharing medication supply and re-allocate inventory	Big data analytics/machine learning/blockchain	^5,54^
	Unexpected increase in demand in short time (C2)	↑	↑	^5,54^
	Many drugs do not have substitutes or substitutes may be less effective (C3)	↑	↑	^5,54^
	Seasonal demand is inaccurate prediction (C4)	Improvement the accuracy of prediction	Big data analytics/machine learning	^7,54^
	Damaged/expired medication (C5)	Implement effective procurement and inventory management systems using technology	Big data analytics	^58–60^

↑Similar to above.

### Risk factor fuzzification

Instead of binary values, fuzzy logic takes into consideration multiple levels of value to address the concept of uncertainty or ambiguity^61^. Since risk assessment in FMEA entails uncertainties from expert judgment, a fuzzification process is used to convert a crisp input into a fuzzy input characterized by a series of fuzzy membership functions^62^. The fuzzification step involves using the fuzzy membership function with implication to evaluate the rules in the rule bases and then aggregating the results of the implication on the rules^52^.

In this study, the input parameters of FMEA risk factors (O-S-D) are fuzzified. During data collection, the risk factors are defined to express the importance of the O-S-D inputs. Based on the experts' recommendations, the corresponding membership functions of each risk factor are defined for all inputs. Following that, a rule set is created by defining various if–then rules based on experts’ opinions to determine the best output formed by various input combinations. Each rule has two main parts: an antecedent (if) and a consequence (then)^63^. In each rule, the antecedent serves as a condition on the inputs to compute the result or output. This step creates a robust structure for applying experts’ viewpoints on the input variables for performing fuzzy inference^52^. An inference is implemented on the input of risk variables based on the fuzzy operators. An FIS is designed independently for each risk factor. Initially, fuzzy propositions are presented using the implication operator during the inference phase^64^.

**Figure 3:**
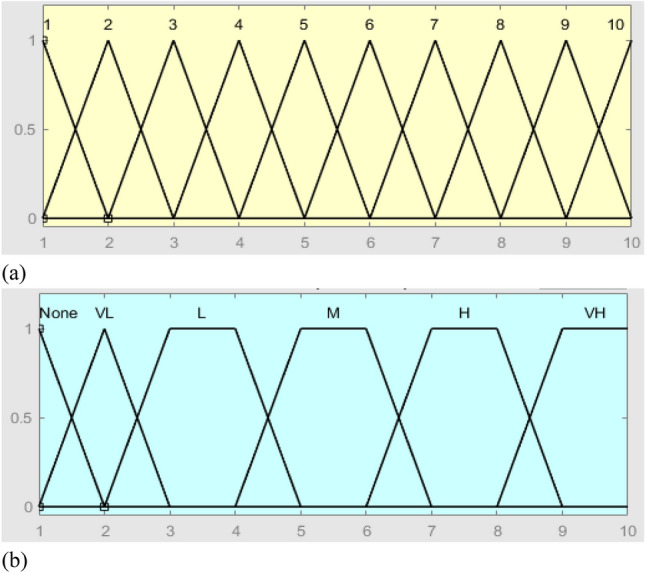
The membership functions of O-factor, (**a**) = input and (**b**) = output.

**Figure 4:**
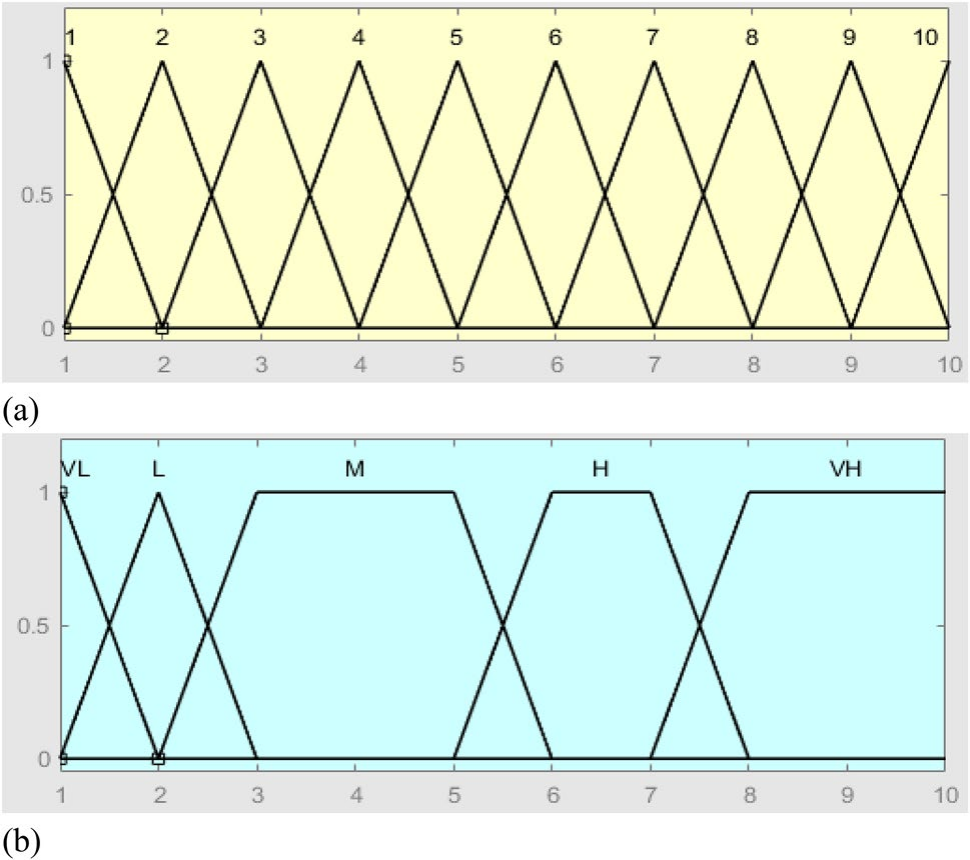
The membership functions of S-factor, (**a**) = input and (**b**) = output.

**Figure 5:**
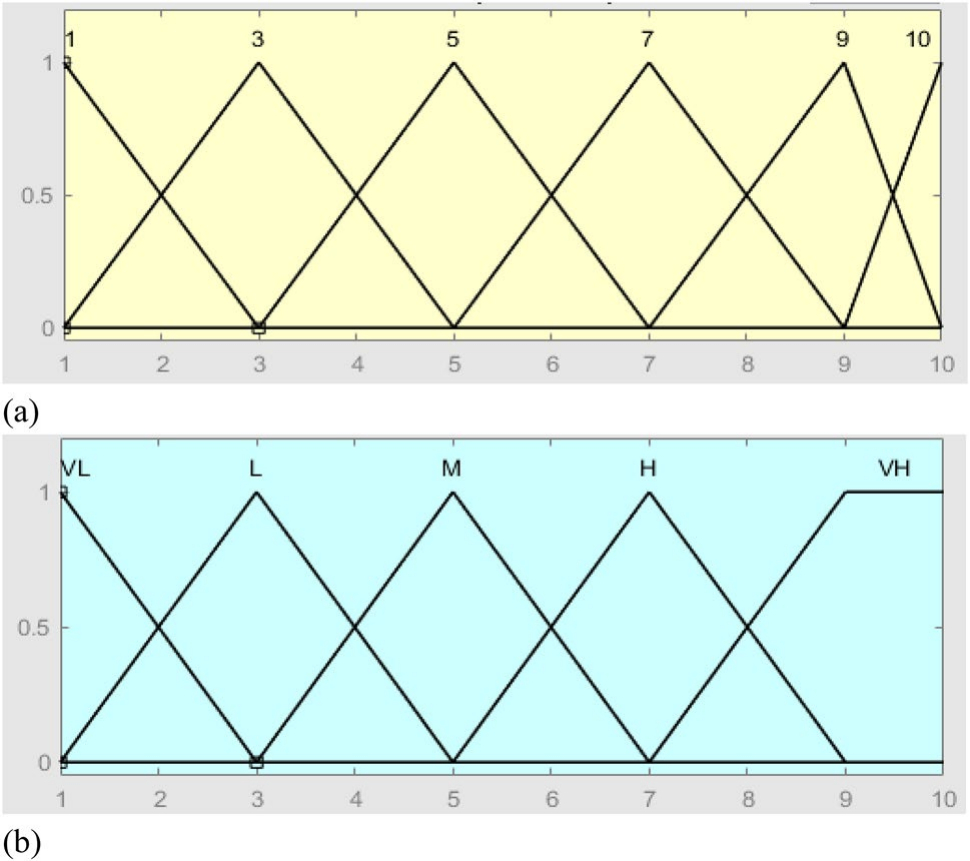
The membership functions of D-factor, (**a**) = input and (**b**) = output.

The FIS inputs (i.e., O-S-D risk factors) consist of experts’ viewpoints while the output consists of judgment on the O-S-D factors. In the FIS, the *triangular membership* functions are defined for the inputs, while the *trapezoidal membership* functions are defined for the output. As shown in Tables 4, 5, 6 and Figs. 3, 4, 5, the membership function outputs are used for the inference engine with fuzzy if–then rules. Using the FIS toolbox in MATLAB (R2020b), a total of 10 rules for the FIS are defined based on the number of input levels of the O and S factors, along with 6 rules of the D factor.

**Table 4:** Fuzzification of O-factor.

Input	Fuzzy of input	Output	Fuzzy of output	Rule
1	1-1-2	None	1-1-2	If Input is 1 then Output is None
2	1-2-3	Very low (VL)	1-2-3	If Input is 2 then Output is VL
3	2-3-4	Low (L)	2-3-4-5	If Input is 3 then Output is L
4	2-4-5	Moderate (M)	4-5-6-7	If Input is 4 then Output is L
5	4-5-6	High (L)	6-7-8-9	If Input is 5 then Output is M
6	5-6-7	Very high (VH)	8-9-10-10	If Input is 6 then Output is M
7	6-7-8			If Input is 7 then Output is H
8	7-8-9			If Input is 8 then Output is H
9	8-9-10			If Input is 9 then Output is VH
10	9-10-10			If Input is 10 then Output is VH

**Table 5:** Fuzzification of S-factor.

Input	Fuzzy of input	Output	Fuzzy of output	Rule
1	1-1-2	Very low (VL)	1-1-2	If Input is 1 then Output is VL
2	1-2-3	Low (L)	1-2-3	If Input is 2 then Output is L
3	2-3-4	Moderate (M)	2-3-5-6	If Input is 3 then Output is M
4	2-4-5	High (L)	5-6-7-8	If Input is 4 then Output is M
5	4-5-6	Very high (VH)	7-8-10-10	If Input is 5 then Output is M
6	5-6-7			If Input is 6 then Output is H
7	6-7-8			If Input is 7 then Output is H
8	7-8-9			If Input is 8 then Output is VH
9	8-9-10			If Input is 9 then Output is VH
10	9-10-10			If Input is 10 then Output is VH

**Table 6:** Fuzzification of D-factor.

Input	Fuzzy of input	Output	Fuzzy of output	Rule
1	1-1-3	Very low (VL)	1-1-3	If Input is 1 then Output is VL
3	1-3-5	Low (L)	1-3-5	If Input is 3 then Output is L
5	3-5-7	Moderate (M)	3-5-7	If Input is 5 then Output is M
7	5-7-9	High (L)	5-7-9	If Input is 7 then Output is H
9	7-9-10	Very high (VH)	7-9-10-10	If Input is 9 then Output is VH
10	9-10-10			If Input is 10 then Output is VH

### Risk factor defuzzification

**Figure 6:**
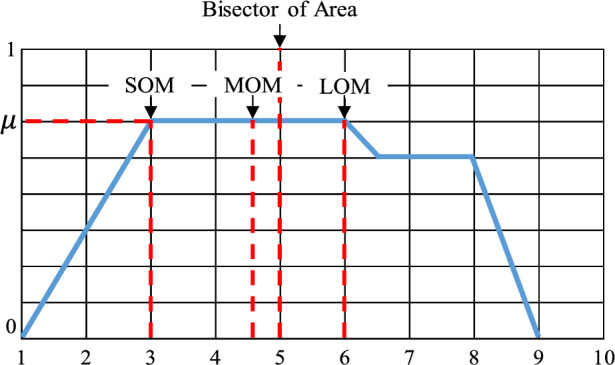
An example of defuzzification methods of SOM, MOM, and LOM.

Defuzzification is used to convert the fuzzy outputs to crisp outputs after the inference process. In defuzzification, various methods for approximating fuzzy outputs to non-fuzzy values are available^65^. The Mamdani type is used in this study. The FIS outputs are calculated using three defuzzification methods to determine the best defuzzification result^66^. They are the Smallest of Maximum (SOM), Middle of Maximum (MOM) and Largest of Maximum (LOM) (see Fig. 6).

According to^67^ and^52^, SOM, MOM, and LOM can be selected from a fuzzy set of outputs, as follows:

1 $$SOM=\underset{u}{{\text{sup}}}\left\{u\in U:{\mu }^{cnceqe}\left(u\right)=\underset{u}{{\text{max}}}\left\{{\mu }^{cnceqe}(u)\right\}\right\}$$

2 $$LOM=\underset{u}{{\text{inf}}}\left\{u\in U:{\mu }^{cnceqe}\left(u\right)=\underset{u}{{\text{max}}}\left\{{\mu }^{cnceqe}(u)\right\}\right\}$$

3 $$MOM=\frac{SOM+LOM}{2}$$

where $\underset{u}{{\text{sup}}}$ and $\underset{u}{{\text{inf}}}$ are the lower bound (LB) and upper bound (UB), ${\mu }^{cnceqe}$ is the membership function of fuzzy set $u$, and $U$ is the range of possible output values. Corresponding to the number of risk factors, three values of SOM, MOM and LOM are calculated. According to the proposed method, each potential risk is tagged with three values computed for each O-S-D factor.

### The fuzzy numbers of risk factor

The FIS toolbox in MATLAB (R2020b) is used to process the fuzzy inputs based on fuzzification of O-S-D factors. Then, the SOM, MOM, and LOM output scores are computed based on the possible number of crisp values of each factor. The results are shown in Table 7.

**Table 7:** The SOM, MOM, and LOM scores of risk factors.

Risk factor											
Occurrence				Severity				Detection			
Crisp	SOM	MOM	LOM	Crisp	SOM	MOM	LOM	Crisp	SOM	MOM	LOM
1	1	1	1	1	1	1	1	1	1	1	1
2	1.99	1.99	1.99	2	1.99	1.99	1.99	–	–	–	–
3	3.07	3.52	3.97	3	3.07	4.01	4.96	3	2.98	2.98	2.98
4	3.07	3.52	3.97	4	3.07	4.01	4.96	–	–	–	–
5	5.05	5.5	5.95	5	3.07	4.01	4.96	5	4.96	4.96	4.96
6	5.05	5.5	5.95	6	6.04	6.49	6.94	–	–	–	–
7	7.03	7.48	7.93	7	6.04	6.49	6.94	7	7.03	7.03	7.03
8	7.03	7.48	7.93	8	8.02	9.01	10	–	–	–	–
9	9.01	9.5	10	9	8.02	9.01	10	9	9.01	9.5	10
10	9.01	9.5	10	10	8.02	9.01	10	10	9.01	9.5	10

### DEA and the cross efficiency method

We use the input-oriented DEA model in this study to illustrate the CRS (Constant Returns to Scale) method of^68^,

4 $${\text{min}}{\theta }_{0}-\varepsilon (\sum_{i=1}^{u}{s}_{i}^{-}+\sum_{r=1}^{v}{s}_{r}^{+})$$

$${\text{s}}.{\text{t}}.\sum_{j=1}^{n}{\lambda }_{j}{x}_{ij}+{s}_{i}^{-}={\theta }_{0}{x}_{i0},i=1, 2, \dots , m,$$

$$\sum_{j=1}^{n}{\lambda }_{j}{y}_{rj}-{s}_{r}^{+}={y}_{r0},r=1, 2, \dots , s,$$

$${\theta }_{0},{\lambda }_{j},{s}_{i}^{-},{s}_{r}^{+} \ge 0,$$

 

Where $n$ denotes the number of DMUs with index $j$. Parameters $m$ and $s$ represent the input ${(x}_{i})$ and output ${(y}_{r})$ numbers. Moreover, ${s}_{i}^{-}$ and ${s}_{r}^{+}$ are the slacks in the input and output. A DMU converts the inputs to outputs in DEA. Its efficiency can be measured using a productivity-related output-to-input ratio^69^. For evaluation, a set of DMUs is used. Each DMU has some managerial discretion in decision-making.

When a standard FMEA model is performed on the DEA, *the failure modes correspond to the DMUs*, while the inputs (*O-S-D) correspond to multiple inputs* of the DEA. Furthermore, multiplying O, S and D results in the RPN (i.e., the FMEA output). The limitations of crisp RPN scores in a standard FMEA model have been highlighted, such as the simple mathematical formula used to compute the RPN can lead to non-intuitive statistical properties^70^. Furthermore, the RPN does not take into account both direct and indirect relationships between each failure mode, and is insufficient to deal with systems or processes with a large number of subsystems and/or components^71^. *DEA can be exploited to tackle the mathematical formula issues of RPN computation,* as it *can handle risk factor weights and consider direct and indirect relationships between the failure modes*^72^.

Because the RPN does not match the DEA output, previous research studies have proposed using any DEA model without outputs, or constant outputs equal to one, when applying DEA to FMEA to achieve an efficiency score for risk prioritization, instead of the traditional RPN score^52,70,73,74^. Traditional DEA methods, however, have some limitations, such as a low discriminating power in efficiency evaluation. *The cross-efficiency method strengthens the discriminatory power of DEA*^75^. Thus, this study employs the DEA cross-efficiency method to improve FMEA for risk analysis.

The traditional DEA model is expanded in two stages in the cross-efficiency method, including self- and peer-evaluation. This addition assesses the overall performance within each DMU by taking into account not only its individual weights but also the weights of all DMUs^76^. To self-evaluate, Eq. (4) is used, where DMU $j$ is evaluated using its extremely favorable weights. In addition, ${\mu }_{rj}$ and ${\nu }_{ij}$ are the optimal output and input weights of the self-evaluation stage for a given DMU $j$ $(j\in N)$, respectively. It is easily demonstrated during the peer-evaluation stage that by using the cross-efficiency method, all DMUs are evaluated using a similar set of weights. Indeed, the $j$ th DMU value of cross-efficiency $({CE}_{j})$ can be computed using Eq. (5) ^77^:

5 $${CE}_{j}=\frac{1}{N}\sum_{ k=1}^{N}{E}_{jk}=\frac{1}{N}\sum_{k=1}^{N}(\frac{{\mu }_{1}{y}_{1jk}{+\mu }_{2}{y}_{2jk}+\dots {+\mu }_{r}{y}_{rjk}}{{\nu }_{1}{x}_{1jk}{+\nu }_{2}{x}_{2jk}+\dots {+\nu }_{m}{x}_{mjk}})$$

For DMU $j$ (${E}_{jk}$) to obtain the cross-efficiency scores of all DMUs, Eq. (5) must be solved $k$ times, each time for a target efficiency score. As shown in Table 8, all the scores can be displayed as a $j\times k$ cross-efficiency matric, with the diagonal part displaying the CRS-efficiency scores ${E}_{jk}^{*}$^76^.

**Table 8:** Cross-efficiency matric of the DMUs.

DMU	Target DMU				Cross-efficiency ${(CE}_{j})$
	${DMU}_{1}$	${DMU}_{2}$	…	${DMU}_{j}$	
${DMU}_{1}$	${E}_{11}^{*}$	${E}_{12}$	…	${E}_{1k}$	${CE}_{1}=\frac{1}{N}\sum_{k=1}^{N}{E}_{1k}$
${DMU}_{2}$	${E}_{21}$	${E}_{22}^{*}$	…	${E}_{2k}$	${CE}_{2}=\frac{1}{N}\sum_{k=1}^{N}{E}_{2k}$
⋮	⋮	⋮	⋮	⋮	⋮
${DMU}_{j}$	${E}_{j1}$	${E}_{j2}$	…	${E}_{jk}^{*}$	${CE}_{j}=\frac{1}{N}\sum_{j, k=1}^{N}{E}_{jk}$

Source: (Liu, Song, and Yang 2019).

## Results, analysis and discussion

**Figure 7:**
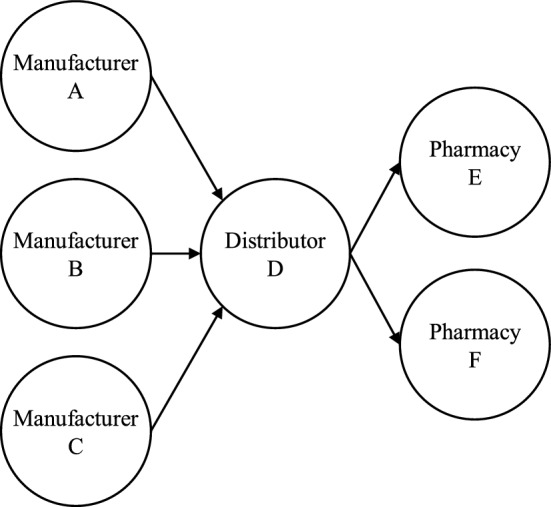
The supply chain structure of the case study.

The developed model is applied to a case study of firms supporting a pharmaceutical supply network in Malaysia. Figure 7 depicts the supply chain structure of the study. This diagram depicts three drug manufacturers (A, B, and C), one distributor (D), and two community pharmacies (E and F). Semi-structured questionnaires designed based on the risk metric of each node (Tables A to C in Appendix) are used during the interview with an expert from the top management of each firm (plant manager, logistics manager, or shop manager). The data collected are analyzed, and the outcomes of the risk assessment, including individual nodes and overall supply chain results, are discussed in the following subsections.

### The risk of the manufacturer node

**Figure 8:**
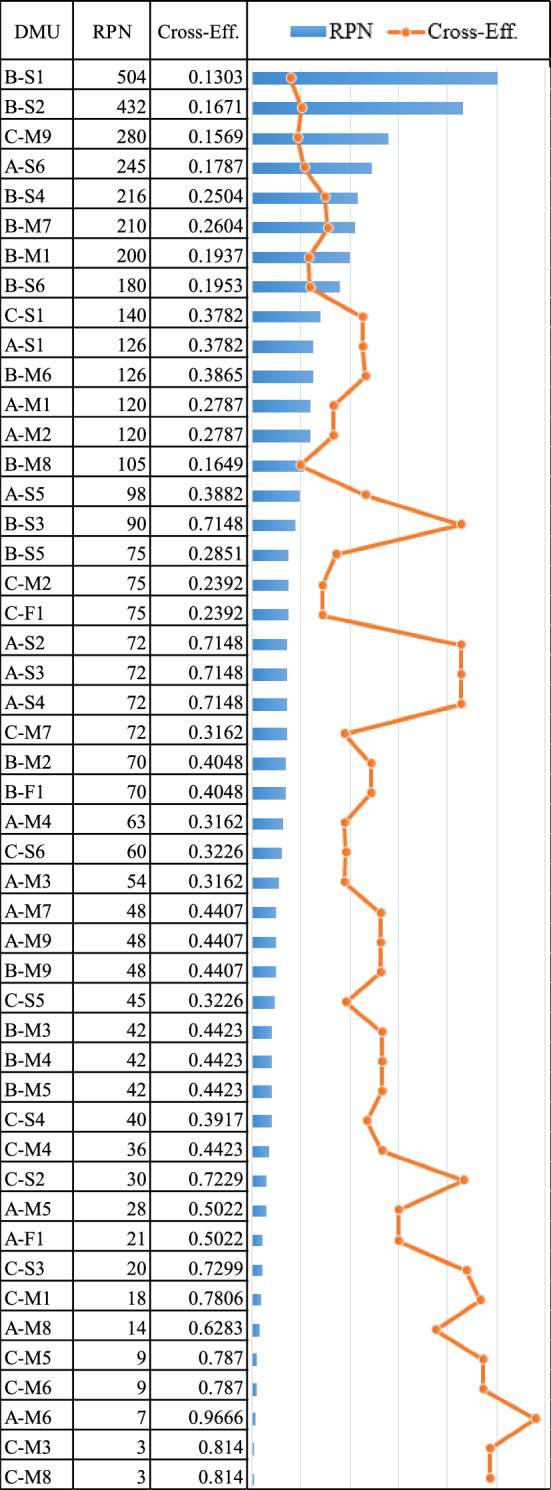
The cross-efficiency of manufacturer node.

For the manufacturer node, the DEA cross-efficiency method (Eq. (5)) is used, as shown in Table 9 and Fig. 8. Because this indicator is expected to be as low as possible when a constant output is considered, all three fuzzy risk factors (O-S-D) of SOM, MOM, and LOM as in Table 7 are considered as the inputs. The output is set to one, and this value is shared by all DMUs. All risk events are dependent on the three manufacturers producing the DMUs in the DEA cross-efficiency method, totaling 48 DMUs.

**Table 9:** The cross-efficiency of manufacturer node.

DMU	Occurrence			Severity			Detection			Cross-Eff	Avg. Cross-Eff
	SOM	MOM	LOM	SOM	MOM	LOM	SOM	MOM	LOM		
A-S1	1.99	1.99	1.99	6.04	6.49	6.94	9.01	9.5	10	0.3782	0.485
A-S2	1	1	1	8.02	9.01	10	9.01	9.5	10	0.7148	
A-S3	1	1	1	8.02	9.01	10	9.01	9.5	10	0.7148	
A-S4	1	1	1	8.02	9.01	10	9.01	9.5	10	0.7148	
A-S5	1.99	1.99	1.99	6.04	6.49	6.94	7.03	7.03	7.03	0.3882	
A-S6	5.05	5.5	5.95	6.04	6.49	6.94	7.03	7.03	7.03	0.1787	
A-M1	3.07	3.52	3.97	6.04	6.49	6.94	4.96	4.96	4.96	0.2787	
A-M2	3.07	3.52	3.97	6.04	6.49	6.94	4.96	4.96	4.96	0.2787	
A-M3	3.07	3.52	3.97	6.04	6.49	6.94	2.98	2.98	2.98	0.3162	
A-M4	3.07	3.52	3.97	6.04	6.49	6.94	2.98	2.98	2.98	0.3162	
A-M5	3.07	3.52	3.97	6.04	6.49	6.94	1	1	1	0.5022	
A-M6	1	1	1	6.04	6.49	6.94	1	1	1	0.9666	
A-M7	1.99	1.99	1.99	8.02	9.01	10	2.98	2.98	2.98	0.4407	
A-M8	1.99	1.99	1.99	6.04	6.49	6.94	1	1	1	0.6283	
A-M9	1.99	1.99	1.99	8.02	9.01	10	2.98	2.98	2.98	0.4407	
A-F1	3.07	3.52	3.97	6.04	6.49	6.94	1	1	1	0.5022	
B-S1	7.03	7.48	7.93	8.02	9.01	10	9.01	9.5	10	0.1303	0.3329
B-S2	5.05	5.5	5.95	8.02	9.01	10	9.01	9.5	10	0.1671	
B-S3	1	1	1	8.02	9.01	10	9.01	9.5	10	0.7148	
B-S4	3.07	3.52	3.97	8.02	9.01	10	9.01	9.5	10	0.2504	
B-S5	3.07	3.52	3.97	3.07	4.01	4.96	4.96	4.96	4.96	0.2851	
B-S6	5.05	5.5	5.95	6.04	6.49	6.94	4.96	4.96	4.96	0.1953	
B-M1	5.05	5.5	5.95	8.02	9.01	10	4.96	4.96	4.96	0.1937	
B-M2	1.99	1.99	1.99	6.04	6.49	6.94	4.96	4.96	4.96	0.4048	
B-M3	1.99	1.99	1.99	6.04	6.49	6.94	2.98	2.98	2.98	0.4423	
B-M4	1.99	1.99	1.99	6.04	6.49	6.94	2.98	2.98	2.98	0.4423	
B-M5	1.99	1.99	1.99	6.04	6.49	6.94	2.98	2.98	2.98	0.4423	
B-M6	1.99	1.99	1.99	8.02	9.01	10	7.03	7.03	7.03	0.3865	
B-M7	3.07	3.52	3.97	8.02	9.01	10	7.03	7.03	7.03	0.2604	
B-M8	7.03	7.48	7.93	3.07	4.01	4.96	4.96	4.96	4.96	0.1649	
B-M9	1.99	1.99	1.99	8.02	9.01	10	2.98	2.98	2.98	0.4407	
B-F1	1.99	1.99	1.99	6.04	6.49	6.94	4.96	4.96	4.96	0.4048	
C-S1	1.99	1.99	1.99	6.04	6.49	6.94	9.01	9.5	10	0.3782	0.5153
C-S2	1	1	1	3.07	4.01	4.96	9.01	9.5	10	0.7229	
C-S3	1	1	1	1.99	1.99	1.99	9.01	9.5	10	0.7299	
C-S4	1.99	1.99	1.99	1.99	1.99	1.99	9.01	9.5	10	0.3917	
C-S5	3.07	3.52	3.97	3.07	4.01	4.96	2.98	2.98	2.98	0.3226	
C-S6	3.07	3.52	3.97	3.07	4.01	4.96	2.98	2.98	2.98	0.3226	
C-M1	1	1	1	6.04	6.49	6.94	2.98	2.98	2.98	0.7806	
C-M2	5.05	5.5	5.95	3.07	4.01	4.96	2.98	2.98	2.98	0.2392	
C-M3	1	1	1	1	1	1	2.98	2.98	2.98	0.814	
C-M4	1.99	1.99	1.99	6.04	6.49	6.94	2.98	2.98	2.98	0.4423	
C-M5	1	1	1	3.07	4.01	4.96	2.98	2.98	2.98	0.787	
C-M6	1	1	1	3.07	4.01	4.96	2.98	2.98	2.98	0.787	
C-M7	3.07	3.52	3.97	6.04	6.49	6.94	2.98	2.98	2.98	0.3162	
C-M8	1	1	1	1	1	1	2.98	2.98	2.98	0.814	
C-M9	7.03	7.48	7.93	8.02	9.01	10	4.96	4.96	4.96	0.1569	
C-F1	5.05	5.5	5.95	3.07	4.01	4.96	2.98	2.98	2.98	0.2392	
Lower	1	1	1	1	1	1	1	1	1	1	-
Upper	9.01	9.5	10	8.02	9.01	10	9.01	9.5	10	0.1094	-

The RPN is employed to determine the relative risk for each failure mode. It is a standard metric for assessing the relative risk of failure modes^78^. To maintain this perspective, we add dummy DMUs of the lower and upper ranks when analyzing the efficiency score. This technique is suitable for a study with multiple nodes or organizations, such as a supply chain, because we analyze different node groups at different times. It provides appropriate results when comparing different node groups because the dummy DMUs run the standard metric.

The results of a comparison between the DEA cross-efficiency and the traditional RPN are presented. When the DEA is used, a low RPN is usually associated with a high-efficiency score. However, as shown in Fig. 8, the efficiency scores of DMUs using this method do not entirely rely on the RPN value (as indicated by the fluctuation of the line chart). This phenomenon suggests that the DEA result is more reasonable in risk prioritzing because it does not adhere to the RPN result, which is still based on a simple mathematical formula, i.e., multiplication of the S, O, and D scores^75^. When the value of cross-efficiency scores concerning each manufacture is prioritized (Table 9), B yields an average efficiency score of 0.3329, which is at the highest risk level. This is followed by A and C, with 0.485 and 0.5153 average efficiency scores, respectively. For B, the failure mode of B-S1 (unavailability of raw materials due to a limit or single supplier yield as a material source from a specific source) has the highest risk score of 0.1303. Following that, B-S2 (raw material scarcity as a result of political turmoil) produces a score of 0.1671.

### The risk of distributor node

**Figure 9:**
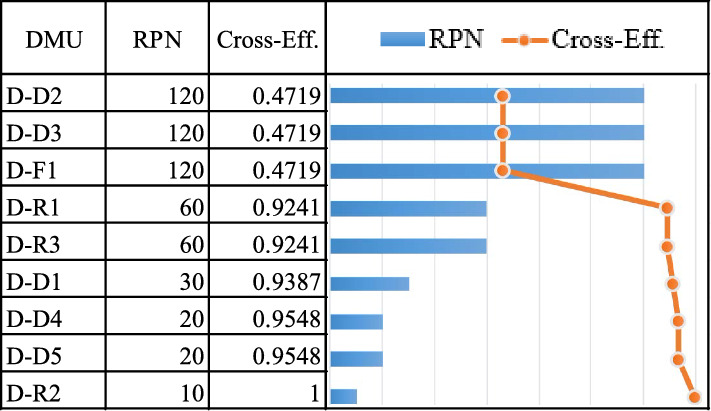
The cross-efficiency of distributor node.

For the distributor, all risk events from a single case study are assigned to DMUs using the DEA cross-efficiency method, yielding a total of 9 DMUs. When the value of cross-efficiency scores is prioritized (Table 10 and Fig. 9), the average efficiency score of distributor D is 0.7902. The failure modes of D-D2 (supply is unable or delayed as a result of transportation and distribution facility failure), D-D3 (supply is unable or delayed due to transportation disruptions), and D-F1 (inability or delay in downstream supply due to inefficiency of just-in-time or lean inventory systems or lack of buffer stock control) have the highest risk, with a score of 0.4719.

**Table 10:** The cross-efficiency of distributor node.

DMU	Occurrence			Severity			Detection			Cross-Eff	Avg. cross-eff
	SOM	MOM	LOM	SOM	MOM	LOM	SOM	MOM	LOM		
D-D1	1	1	1	3.07	4.01	4.96	9.01	9.5	10	0.9387	0.7902
D-D2	1.99	1.99	1.99	6.04	6.49	6.94	9.01	9.5	10	0.4719	
D-D3	1.99	1.99	1.99	6.04	6.49	6.94	9.01	9.5	10	0.4719	
D-D4	1	1	1	1.99	1.99	1.99	9.01	9.5	10	0.9548	
D-D5	1	1	1	1.99	1.99	1.99	9.01	9.5	10	0.9548	
D-F1	1.99	1.99	1.99	6.04	6.49	6.94	9.01	9.5	10	0.4719	
D-R1	1	1	1	6.04	6.49	6.94	9.01	9.5	10	0.9241	
D-R2	1	1	1	1	1	1	9.01	9.5	10	1	
D-R3	1	1	1	6.04	6.49	6.94	9.01	9.5	10	0.9241	
Lower	1	1	1	1	1	1	1	1	1	1	-
Upper	9.01	9.5	10	8.02	9.01	10	9.01	9.5	10	0.1094	-

### The risk of pharmacy node

**Figure 10:**
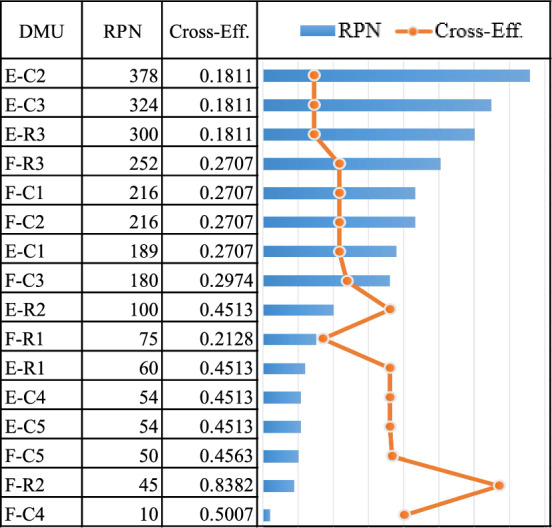
The cross-efficiency of pharmacy node.

The risk event is determined by both pharmacies that comprise the DMUs in the DEA cross-efficiency method, yielding a total of 16 DMUs. When the value of cross-efficiency scores for this node (Table 11 and Fig. 10) concerning each shop is prioritized, E yields an average efficiency score of 0.3274, which is at a higher risk level than that of F. F has an average efficiency score of 0.3897. For E, the failure modes with the highest risk of score of 0.1811 are E-C2 (unavailability of a product for patients due to an unexpected increase in demand in a short period of time), E-C3 (unavailability of a product for patients from many drugs that do not have substitutes or substitutes that are less effective), and E-R3 (upstream supply is unable or delayed because only a few manufacturers produce drugs).

**Table 11:** The cross-efficiency of pharmacy node.

DMU	Occurrence			Severity			Detection			Cross-Eff	Avg. Cross-Eff
	SOM	MOM	LOM	SOM	MOM	LOM	SOM	MOM	LOM		
E-C1	3.07	3.52	3.97	6.04	6.49	6.94	9.01	9.5	10	0.2707	0.3274
E-C2	5.05	5.5	5.95	6.04	6.49	6.94	9.01	9.5	10	0.1811	
E-C3	5.05	5.5	5.95	6.04	6.49	6.94	9.01	9.5	10	0.1811	
E-C4	1.99	1.99	1.99	3.07	4.01	4.96	9.01	9.5	10	0.4513	
E-C5	1.99	1.99	1.99	3.07	4.01	4.96	9.01	9.5	10	0.4513	
E-R1	1.99	1.99	1.99	3.07	4.01	4.96	9.01	9.5	10	0.4513	
E-R2	1.99	1.99	1.99	3.07	4.01	4.96	9.01	9.5	10	0.4513	
E-R3	5.05	5.5	5.95	6.04	6.49	6.94	9.01	9.5	10	0.1811	
F-C1	3.07	3.52	3.97	6.04	6.49	6.94	9.01	9.5	10	0.2707	0.3897
F-C2	3.07	3.52	3.97	6.04	6.49	6.94	9.01	9.5	10	0.2707	
F-C3	3.07	3.52	3.97	3.07	4.01	4.96	9.01	9.5	10	0.2974	
F-C4	1.99	1.99	1.99	3.07	4.01	4.96	1	1	1	0.5007	
F-C5	1.99	1.99	1.99	3.07	4.01	4.96	4.96	4.96	4.96	0.4563	
F-R1	5.05	5.5	5.95	3.07	4.01	4.96	4.96	4.96	4.96	0.2128	
F-R2	1	1	1	3.07	4.01	4.96	9.01	9.5	10	0.8382	
F-R3	3.07	3.52	3.97	6.04	6.49	6.94	9.01	9.5	10	0.2707	
Lower	1	1	1	1	1	1	1	1	1	1	-
Upper	9.01	9.5	10	8.02	9.01	10	9.01	9.5	10	0.1108	-

### The efficiency of supply chain

To describe the overall risk level of the pharmaceutical supply chain, the quantitative values are converted into linguistic variables to describe the risk in words^79^. As a result, the risk scores of cross-efficiency are transferred to the linguistic level for further clarification. Table 12 shows the rank of risk level in this study based on the cross-efficiency score. As an example, if the original value of O-S-D is 5–5-5, the cross-efficiency score of this value is 0.2599, indicating a moderate risk level based on the step explained in the methodology. Overall, there are four levels of risk: low (cross-efficiency score from 0.5 to 1), moderate (cross-efficiency score from 0.25 to less than 0.5), high (cross-efficiency score from 0.125 to less than 0.25), and critical (cross-efficiency score less than 0.125). Table 13 shows the overall risk levels of the pharmaceutical supply chain case study.

**Table 12:**
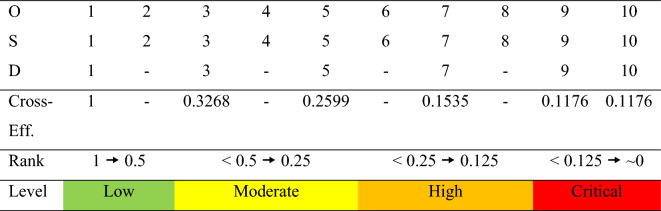
The rank of risk level based on the cross-efficiency score.

**Table 13:**
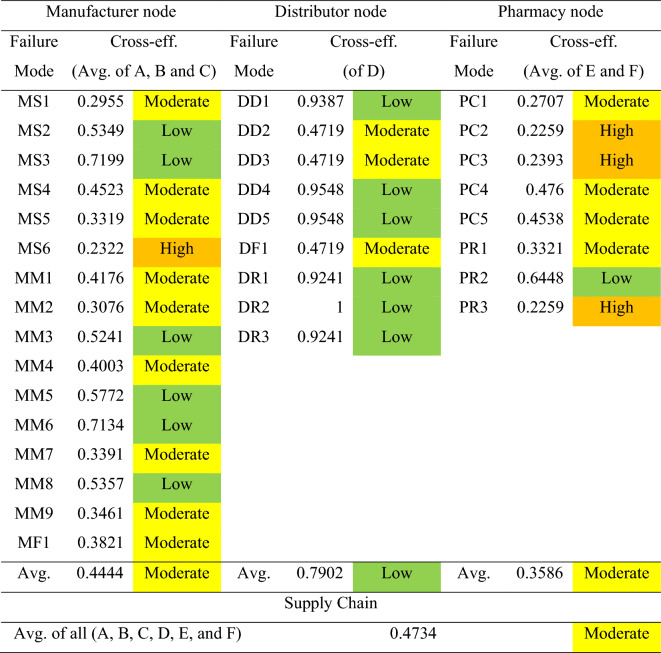
The risk outcome of the pharmaceutical supply chain.

According to Table 13, the average risk of A, B, and C in the manufacturer node is moderate (cross-efficiency score is 0.4444). It is discovered that the failure mode of MS6 (delay in raw material supply due to overseas suppliers) is a high risk. Similarly, developing countries like Malaysia rely heavily on foreign countries such as China, India, and Russia to import various chemicals and other raw materials for the pharmaceutical industry^80^. As a result, natural, man-made and other disasters can have a significant impact on raw material imports.

The average risk level for the distributor node is low (cross-efficiency score is 0.7902). Three items pertaining to the moderate level (cross-efficiency score is 0.4719) are: DD2 (supply is unable or delayed as a result of transportation and distribution facility failure), DD3 (supply is unable or delayed due to transportation disruptions), and DF1 (inability or delay in supplying downstream due to inefficiency or lack of buffer stock control in a just-in-time or lean inventory system). Moreover, drug availability and quality losses during storage and transportation are the most significant challenges of the pharmaceutical supply chain. The transportation and sorting processes affect the delivery time, while breakdowns and uncertainty are the primary issues^81^. Furthermore, transportation disruptions can cause severe effects to the pharmaceutical supply chain operations, necessitating a timely response to such a challenge^82^. This factor is particularly significant in developing countries where drug administration is hampered by a lack of transportation infrastructure and facilities. It can also affect drug delivery accuracy^83^. Pharmaceutical product shortages can be exacerbated by lean inventory management practices. A just-in-time inventory management system is widely used by manufacturers, distribution centers, and healthcare organizations. However, in many cases, it leads to lower inventory levels across the supply chain, which increases the likelihood of shortage occurrence^84^.

The pharmacy node has a moderate risk level (cross-efficiency score is 0.3586). In comparison with other nodes, this node poses the greatest risk. The reason is that it covers three failure modes that fall in the high-risk level, including PC2 (unavailability of a product for patients due to an unexpected increase in demand in a short period), PC3 (unavailability of a product for patients from many drugs that do not have substitutes or substitutes that are less effective), and PR3 (upstream supply is unable or delayed because only a few manufacturers produce drugs). In general, drug shortages can occur for a variety of reasons. As an example, unexpectedly high demand or demand fluctuations can cause drug shortages, which typically affect front-line delivery by pharmacies^85^. Moreover, substitution medicines can help avoid some of the issues that pharmacies face daily, such as the risk of delivery delays, damaged medication, and seller stockouts. Nonetheless, there are only few or no substitute medications available^86^. Additionally, if only few independent manufacturers produce some specialty drugs, these manufacturers can pose a risk to the front-line pharmacy^87^. Correspondingly, the average cross-efficiency score of all nodes in the pharmaceutical supply chain is 0.4734, indicating a moderate level of risk. This average value is appropriate for defining the overall efficiency when using the DEA method in network structures such as supply chains^88^.

The following section will discuss the interaction of digital technologies with the risks factors, along with managerial implication and proposed framework to guide digitalization supply chain for risk mitigation.

### Interaction of digital technologies with risks level

**Figure 11:**
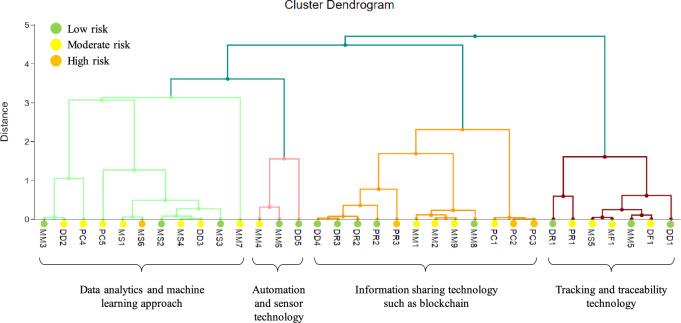
Cluster of Pharma 4.0 main technologies related to the failure mode and its cross-efficiency score using the hierarchical cluster analysis method.

This study further explores the interaction of digital technologies with the risks in the pharmaceutical supply chain. The hierarchical cluster analysis (HCA) method in the DATAtab laboratory (https://datatab.net/) was used to cluster the potential digital technologies related to the risk level of cross-efficiency score. Based on the column mapping to digital technology in Table 3, HCA was exploited to assign the risk events to four primary technologies. Figure 11 shows the cluster dendogram illustrating the interaction between digital technologies and risks level in the pharmaceutical supply chain.

According to Fig. 11, the three high risk events (PR3, PC2 and PC3) are located in the cluster of information sharing technology such as blockchain. Another high risk event (MS6) is situated in the cluster of data analytics and machine learning. Hence, to deal with these various risks events, advanced methodologies and principles must be followed to meet the needs of this complex pharmaceutical supply chain network both internally and externally. Variations in the market economy force the related pharmaceutical firms to change their strategies from time to time. Owing to a constant and fluctuating demand, predicting the correct volume is challenging. Moreover, the time spent at each level of the supply chain is critical in determining supply delivery on time. Practical strategies and methods used by all players to achieve on-time delivery and address product complexity are required^89^. Many useful insights are derived from the digitalization supply chain and the Pharma 4.0 era to develop a conceptual framework for the effective operation of any given pharmaceutical supply chain.

## Managerial implications

Considering that this study's risk assessment of the pharmaceutical supply chain is based on a risk management approach, it is logical to adopt the method of controlling and prioritizing risks within a network of interconnected risks. This approach is instrumental in reducing or mitigating risk exposure effectively. Managing these risks necessitates the utilization of a risk portfolio rather than addressing individual risks in isolation.

In this section, we explore risk mitigation strategies for interdependent risk portfolios by identifying potential technologies with similar marginal contributions. Based on the findings of risk assessment in the case study, the implications on the significant risk level and cluster of potential digital technologies recommendations are presented, as follows.

The collaborative technology for information sharing is significant for the overall players in the pharmaceutical supply network. The main collaboration initiative is to administer a strict inventory control and to let stakeholders know how many products are available, leading to resolving a shortage or reducing the bullwhip effect in the network. To successfully collaborate in a supply chain, various members must agree on mutual goals and synchronize their decisions. By exchanging information in real-time, digitalization has the potential to convert and reshape the pharmaceutical supply chain and improve coordination among supply chain partners. The blockchain technology can bridge the supply chain information such as the inventory visibility gap by improving end-to-end data visibility among the supply chain partners through sharing of backlog information.

In the event of a drug shortage or a lack of substitute drugs (PC2 and PC3), drug sharing or exchange in the pharmacy network can be investigated. Again, the blockchain technology offers an ideal solution. To establish a drug-sharing network based on the blockchain technology, decentralization (i.e., a transparent medium) enables data exchange and recording; thus, entities searching for records in such a credible distributed system could find solid and transparent data on transactions. As a result, securing explicitly open and trustworthy repositories that are required for the drug supply chain in the pharmaceutical business network, where the required data can be easily accessed and tracked by all involved entities, is essential.

Additionally, blockchain technology is advantageous for mitigating the moderate risk of drug shortages due to unpredictable demand (PC1), particularly relevant to PC2 and PC3 (as indicated in Fig. 11). Furthermore, if the blockchain system is tailored to connect with distributors and manufacturers, it can facilitate the reliable transmission of shortage item information to enhance their performance. This improvement includes reducing delivery times, expanding production capacity, and implementing stringent waste control measures, which are associated with the high risk of PR3 and the low risks of DR3 and DD4. Expanding the blockchain network is also valuable for enhancing network coordination, mitigating the low risk of PR2 and DR2, and enabling members to steer clear of unethical or unregulated marketing practices for scarce items.

In the context of data analytics and machine learning, supply chain managers in manufacturing can employ a de-globalization strategy, supported by big data analytics. A recent study by^90^ have noted that the trend toward de-globalization, while resulting in higher costs, can also introduce higher supply volatility due to fewer input sourcing channels. Big data analytics is recognized as a technological pillar that enhances cost competitiveness for onshore production, influences production retention decisions, and aids in the selection of local or regional multi-supliers based on their performance. This strategy mitigated the risk of supply delays from overseas suppliers, particularly the high risk of MS6. It also addresses related global spply chain issues such as the moderate risk of MS4 (trade disputes) and the low risks of MS2 (political turmoil) and MS3 (armed conflicts)..

During application, digital de-globalization is outlined as the state of a digital form that connects regional and national industries, companies, and individuals through digitally enabled or supported flows of data, information, ideas, and knowledge, as well as flows of goods, services, investment, and capital. Big data analytics are common technologies that support such flows, while digitization-enabled platforms like e-commerce and online marketplaces abundantly prompt digital trade and transaction flows that provide a big data source of business. In this context, the moderate risk of MS1, involving limited or single supplier dependence, can be addressed using supply source big data.

Moreover, in pharmacies, AI algorithms can deal with unexpected and unpredictable increases in demand (PC1, PC2 and PC3) in a short time by providing advanced forecasting methods. To forecast trends and obtain optimal models with good accuracy, cutting-edge AI and deep learning algorithms offer viable solutions.

### A Proposed framework to incorporate and encourage digitalization of the pharmaceutical supply chain

Based on the results of the risk assessment in Section "Results, analysis and discussion", it is evident that there are high-risk events within the pharmaceutical supply chain. These high-risk events include: S6 (Delays in raw material supply, primarily associated with the sourcing process); R3 (Delays in supply due to poor order fulfillment and inventory replenishment, which are interconnected processes); C2 and C3 (Unavailability of products in the consumption process).

These risks can be effectively mitigated through the strategic implementation of digital technologies, as discussed in the previous section. For instance, the utilization of big data and data analytics technologies can play a crucial role in mitigating risks associated with sourcing issues. Similarly, the adoption of information sharing technologies can enhance the management of risks related to order fulfillment and inventory replenishment. Furthermore, better demand forecasting in the consumption phase can be achieved through these technologies.

It's important to note that addressing these high-risk events with these technologies can simultaneously help mitigate other corresponding moderate and low-level risks due to the positive side effects of these actions, as illustrated in the previous section on risk portfolio addressing a network of interconnected risks.

**Figure 12:**
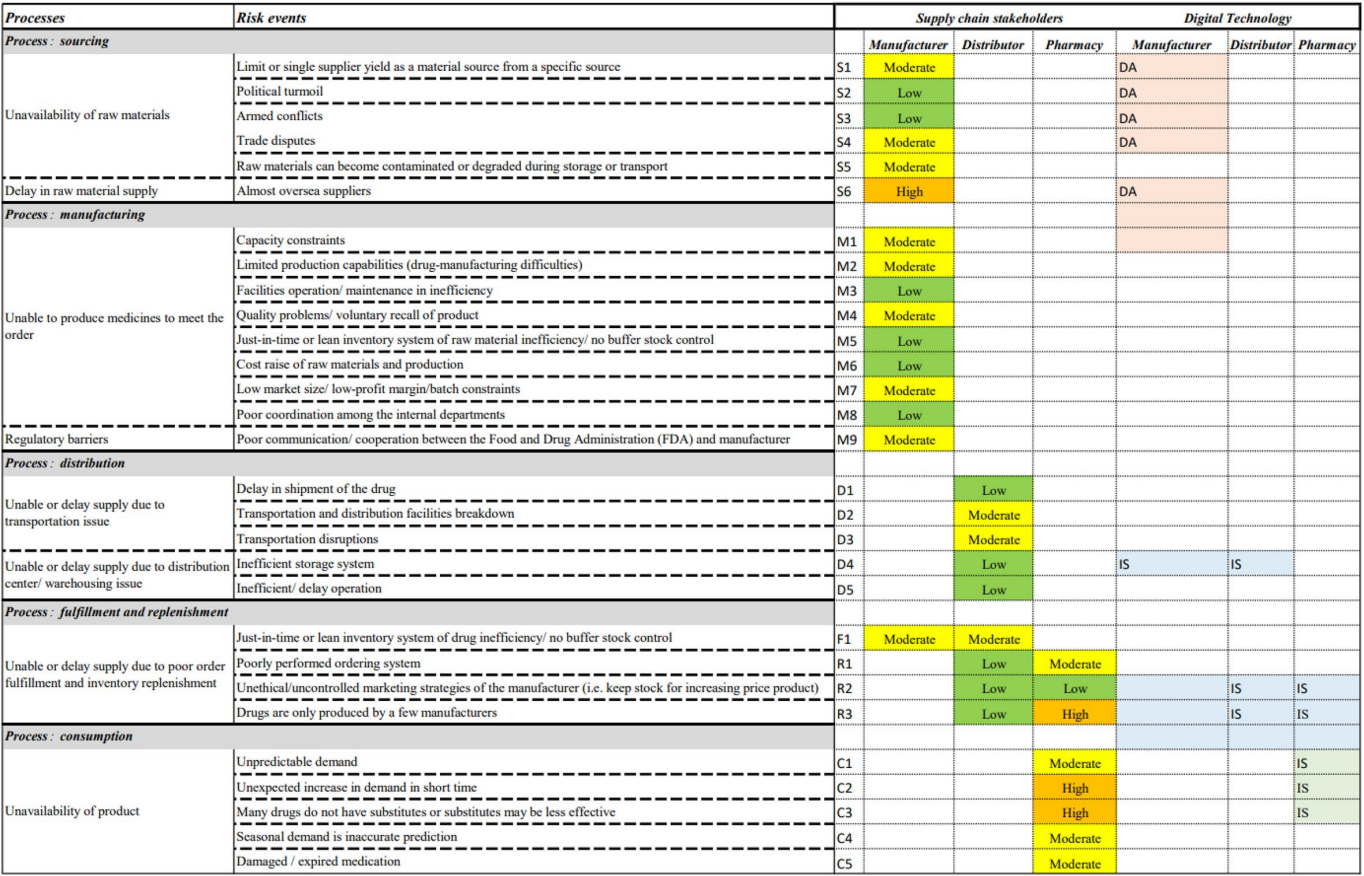
Framework to incorporate digital technologies in the pharmaceutical supply chain.

**Figure 13:**
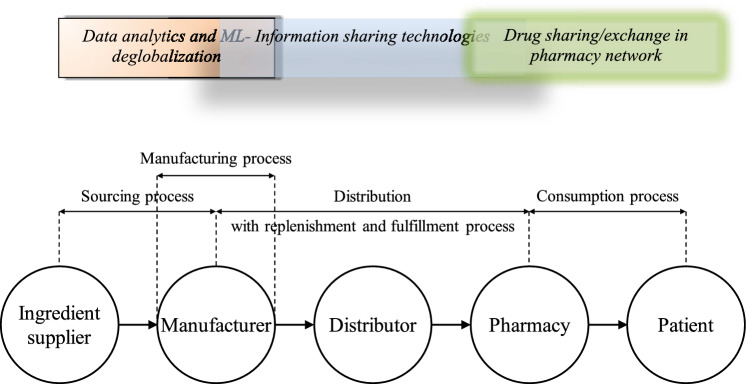
Integration of digital technologies in the pharmaceutical supply chain.

Figure 12 shows the framework on how digital technologies can be harnessed to address risks in the pharmaceutical supply chain. Big Data and data analytics technologies (DA) can effectively mitigate risks in the sourcing and manufacturing processes and should be primarily employed by manufacturers. Information sharing (IS) technologies can significantly reduce risks in the distribution, fulfillment, and replenishment processes. Therefore, a collaborative approach involving manufacturers, distributors, and pharmacies is recommended. Within the consumption process, establishing a drug-sharing (IS) network at both government, community, private and chain pharmacies can be a valuable strategy to mitigate supply shortage risks (Fig. 13).

This framework serves as a guideline for the incorporation of digital technologies into the pharmaceutical supply chain. The implementation of these digital technologies should be a collaborative effort among the various chain members. For instance, instead of creating discrete information-sharing platforms exclusively among pharmacies, it may be beneficial for distributors or manufacturers to take the lead in setting up the infrastructure. This approach can expedite the flow of information upstream, enabling faster responses to address supply shortages.

Furthermore, the proposed framework has the potential to inspire future research and encourage deeper discussions with stakeholders. The framework can be further refined and improved through triangulation and further inputs from all key stakeholder and policy makers.

## Conclusion

Pharmaceutical supply issues or drug shortages are not a new concern; they have long been a serious and growing challenge in the global healthcare system. This is especially true in under-developed or developing countries where drug supplies are limited. In the Pharma 4.0 era, a digital supply chain of pharmaceutical supply processes is critical to improving the overall supply chain performance. In addition, several emerging technologies are beneficial for use in the pharmaceutical supply chain to address supply shortages. This study introduces the concept of risk management for identifying key risk factors in the pharmaceutical supply chain and propose an appropriate digital technology platform for pharmaceutical supply chain management to overcomehis serious issue. This study has contributed to the interaction of technologies in pharmaceutical supply chain performance and provides managerial insights with a proposed framework on how to incorporate and encourage digitalization of the pharmaceutical supply chain for achieving robustness in supply chains using a risk management approach. Through a case study of the pharmaceutical supply chain in Malaysia, this research has discovered that the pharmacy node is the most critical. Shortages arise due to unexpected demand, the same applies to scarcity of specialty or substitute drugs. To address these shortages, this study proposed the implementation of appropriate digital technology platforms for supply chain collaboration, including big data analytics and blockchain technologies.

The study's limitation lies in its focus on a small supply chain, restricting the generalizability to pharmaceutical supply chains in other countries or regions. To address this limitation, future research should encompass larger supply chain networks and diverse geographical contexts to provide a more comprehensive evaluation. Furthermore, enhancing the proposed digital technology integration framework for the pharmaceutical supply chain can be achieved by gathering additional inputs and feedback from key stakeholders.

### Supplementary Information

Supplementary Information.

## Data Availability

The data that support the findings of this study are available from the corresponding author on reasonable request.
