# Supplementary material for: Digitalization enhancement in the pharmaceutical supply network using a supply chain risk management approach
Source: Sci Rep. 2023 Dec 15;13:22287. doi: 10.1038/s41598-023-49606-z (PMC10721629; doi:10.1038/s41598-023-49606-z)
Supplement: Supplementary file 1 — Supplementary Information. [file 41598_2023_49606_MOESM1_ESM.docx]

# Appendix

Table A. The risk assessment metric for manufacturer.

| Failure no. | Event and cause of event: | O | S | Effective of applied of digital-based technology in detecting or preventing the failure: | D |
| --- | --- | --- | --- | --- | --- |
| S1 | Unavailability of raw materials as a result of a limit or single supplier yield as a material source from a specific source. | 🗌 | 🗌 | Using a data analysis tool, such as a big data analytics, for supplier evaluation and selection based on supplier performance and supplier risk of multiple suppliers. | 🗌 |
| S2 | Raw material scarcity as a result of political turmoil | 🗌 | 🗌 |  |  |
| S3 | Raw material scarcity is caused by armed conflicts. | 🗌 | 🗌 |  |  |
| S4 | Raw materials are unavailable due to trade disputes. | 🗌 | 🗌 |  |  |
| S5 | Unavailability of raw materials is caused by raw materials becoming contaminated or degraded during storage or transport. | 🗌 | 🗌 | Promote transporter services capable of developing material tracking and traceability systems, such as cyber-physical systems. | 🗌 |
| S6 | Delay in raw material supply due to overseas suppliers. | 🗌 | 🗌 | Using a data analysis tool for supplier selection to trade-off between globalization and de-globalization of multiple suppliers, such as a big data analytics. | 🗌 |
| M1 | Because of capacity constraints, it is unable to produce enough medicines to meet the demand. | 🗌 | 🗌 | Create a production network based on process analysis technologies and a platform for sharing information or knowledge, such as blockchain. | 🗌 |
| M2 | Unable to produce medicines to meet the order that reason from limited production capabilities (drug-manufacturing difficulties). | 🗌 | 🗌 |  |  |
| M3 | Inability to produce medicines to meet demand due to inefficient facility operation and maintenance. | 🗌 | 🗌 | Improve the efficiency of manufacturing processes by combining automation and sensors with process analytical technologies, as well as learning from production data using artificial intelligence such as machine learning. | 🗌 |
| M4 | Inability to produce medicines to meet orders due to quality issues or voluntary product recall. | 🗌 | 🗌 | Create more robust and agile manufacturing processes, promote higher levels of quality management, and use automation and sensor systems with process analytical technologies as a sample. | 🗌 |
| M5 | Unable to produce medicines in time to meet orders due to raw material inefficiency and a lack of buffer stock control in a just-in-time or lean inventory system. | 🗌 | 🗌 | Improve the material inventory and control management system based on inventory tracking and traceability systems such as the cyber-physical system. | 🗌 |
| M6 | Unable to produce medicines to meet orders due to raw material and production cost increases. | 🗌 | 🗌 | Increase production efficiency and quality by utilizing automation and sensor systems with process analytical technologies that can support total cost reduction. | 🗌 |
| M7 | Inability to produce medicines to meet orders due to a small market size, a low profit margin, or batch constraints. | 🗌 | 🗌 | Create a flexible small-scale production solution or customized manufacturing technology, such as 3D printing. | 🗌 |
| M8 | Inability to produce medicines in time to meet the order due to poor coordination among internal departments. | 🗌 | 🗌 | Use an information sharing platform such as blockchain to improve the information transfer process. | 🗌 |
| M9 | Poor communication and cooperation between the Food and Drug Administration (FDA) and the manufacturer create regulatory barriers. | 🗌 | 🗌 | Use an information sharing platform such as blockchain to develop or support a transparent communication and cooperation system. | 🗌 |
| F1 | Inability or delay in downstream supply due to inefficiency of just-in-time or lean inventory systems or lack of buffer stock control. | 🗌 | 🗌 | Create a system for tracking and tracing materials, such as a cyber-physical system. | 🗌 |

Table B. The risk assessment metric for distributor.

| Failure no. | Event and cause of event: | O | S | Effective of applied of digital-based technology in detecting or preventing the failure: | D |
| --- | --- | --- | --- | --- | --- |
| D1 | Supply is unable or delayed as a result of a delay in the shipment of the drug from the transportation operation. | 🗌 | 🗌 | Create a product shipment tracking and traceability system that can be used in transportation, such as a cyber-physical system, the internet of things, cloud computing, RFID, and auto-ID. | 🗌 |
| D2 | Supply is unable or delayed as a result of transportation and distribution facility failure. | 🗌 | 🗌 | Enhance the efficiency of transportation operations through big data analytics and learning from operational data through artificial intelligence such as machine learning. | 🗌 |
| D3 | Supply is unable or delayed due to transportation disruptions. | 🗌 | 🗌 | As an example, conduct a feasibility study on changing modes of transportation, routing optimization, and drone earing technology. | 🗌 |
| D4 | Supply is unable or delayed due to an inefficient storage system. | 🗌 | 🗌 | Enhance the efficiency of storage operations through big data analytics and inventory tracking and traceability systems for product conditions storage, such as blockchain and IoT integration. | 🗌 |
| D5 | Supply is unable or delayed as a result of inefficient or delayed in warehouse operation. | 🗌 | 🗌 | Increase the efficiency of warehouse operations by combining automation and sensor systems with process analytical technologies. | 🗌 |
| F1 | Inability or delay in supplying downstream due to inefficiency or lack of buffer stock control in a just-in-time or lean inventory system. | 🗌 | 🗌 | Create a system for tracking and tracing product inventory, such as a cyber-physical system. | 🗌 |
| R1 | Supply from upstream is unavailable or delayed as a result of a poorly performed ordering system. | 🗌 | 🗌 | Create a system for ordering practices that makes use of cloud and fog computing, cyber-physical systems, e-procurement, and open contracting, among other things. | 🗌 |
| R2 | Inability or delay in upstream supply due to unethical or uncontrolled marketing strategies employed by the manufacturer. | 🗌 | 🗌 | Intensive inventory control with big data analytics and promotion of the system that allows stakeholders to know how much product is available with blockchain. | 🗌 |
| R3 | Upstream supply is unable or delayed because only a few manufacturers produce drugs. | 🗌 | 🗌 |  |  |

Table C. The risk assessment metric for pharmacy.

| Failure no. | Event and cause of event: | O | S | Effective of applied of digital-based technology in detecting or preventing the failure: | D |
| --- | --- | --- | --- | --- | --- |
| C1 | Unpredictable demand causes product shortages for patients. | 🗌 | 🗌 | Promote the independent pharmacies should coordinate and work together, closely to be informed, and sharing medication supply and re-allocating inventory using big data analytics, machine learning, and blockchain, among other things. | 🗌 |
| C2 | Unavailability of a product for patients due to an unexpected increase in demand in a short period of time. | 🗌 | 🗌 |  |  |
| C3 | Unavailability of product for patients from many drugs that do not have substitutes or substitutes that are less effective. | 🗌 | 🗌 |  |  |
| C4 | Product unavailability due to seasonal demand is an inaccurate prediction. | 🗌 | 🗌 | Improve prediction accuracy with big data analytics and machine learning technology. | 🗌 |
| C5 | Product is unavailable due to damaged or expired medication. | 🗌 | 🗌 | Implementation of effective procurement and inventory management systems utilizing big data analytics and cyber-physical systems technology. | 🗌 |
| R1 | Supply from upstream is unavailable or delayed as a result of a poorly performed ordering system. | 🗌 | 🗌 | Create a system for ordering practices that makes use of cloud and fog computing, cyber-physical systems, e-procurement, and open contracting, among other things. | 🗌 |
| R2 | Inability or delay in upstream supply due to unethical or uncontrolled marketing strategies employed by the manufacturer. | 🗌 | 🗌 | Intensive inventory control with big data analytics and promotion of the system that allows stakeholders to know how much product is available with blockchain. | 🗌 |
| R3 | Upstream supply is unable or delayed because only a few manufacturers produce drugs. | 🗌 | 🗌 |  |  |
